# Supplementary material for: tDCS induced GABA change is associated with the simulated electric field in M1, an effect mediated by grey matter volume in the MRS voxel
Source: Brain Stimul. Author manuscript; Available in PMC 2022 Oct 5. (PMC7613675; doi:10.1016/j.brs.2022.07.049)
Supplement: Supplementary data [file EMS154971-supplement-Supplementary_data.docx]

**Supplementary Material**

**
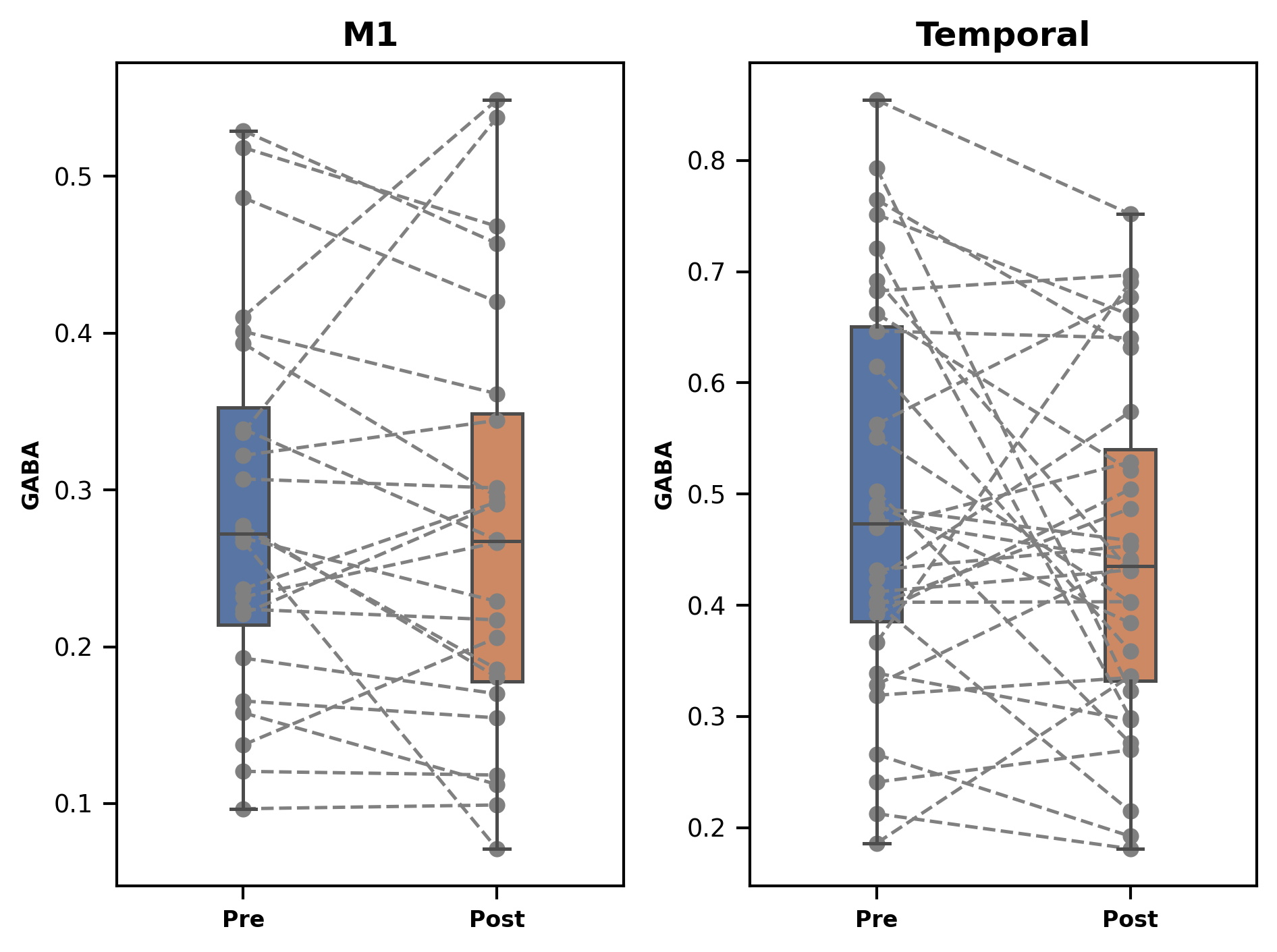
**

*Supplementary figure 1: Pre and post GABA values for the motor region (left) and the temporal region (right).*

**
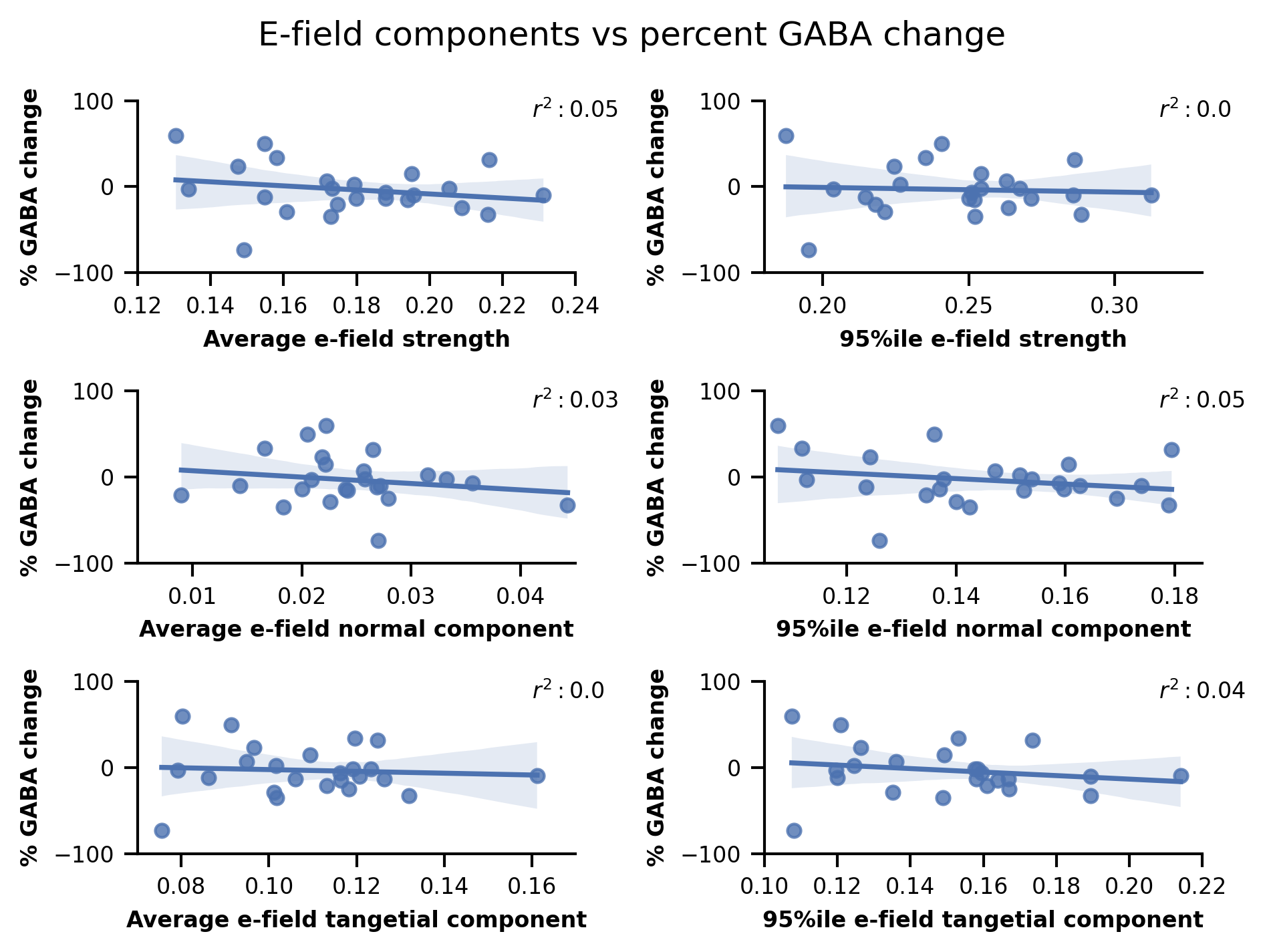
**

*Supplementary figure 2: Association between all the E-field estimates (magnitude, normal and tangent; mean and 95th percentile), in the M1 MRS voxel, and the percent GABA change.*

**
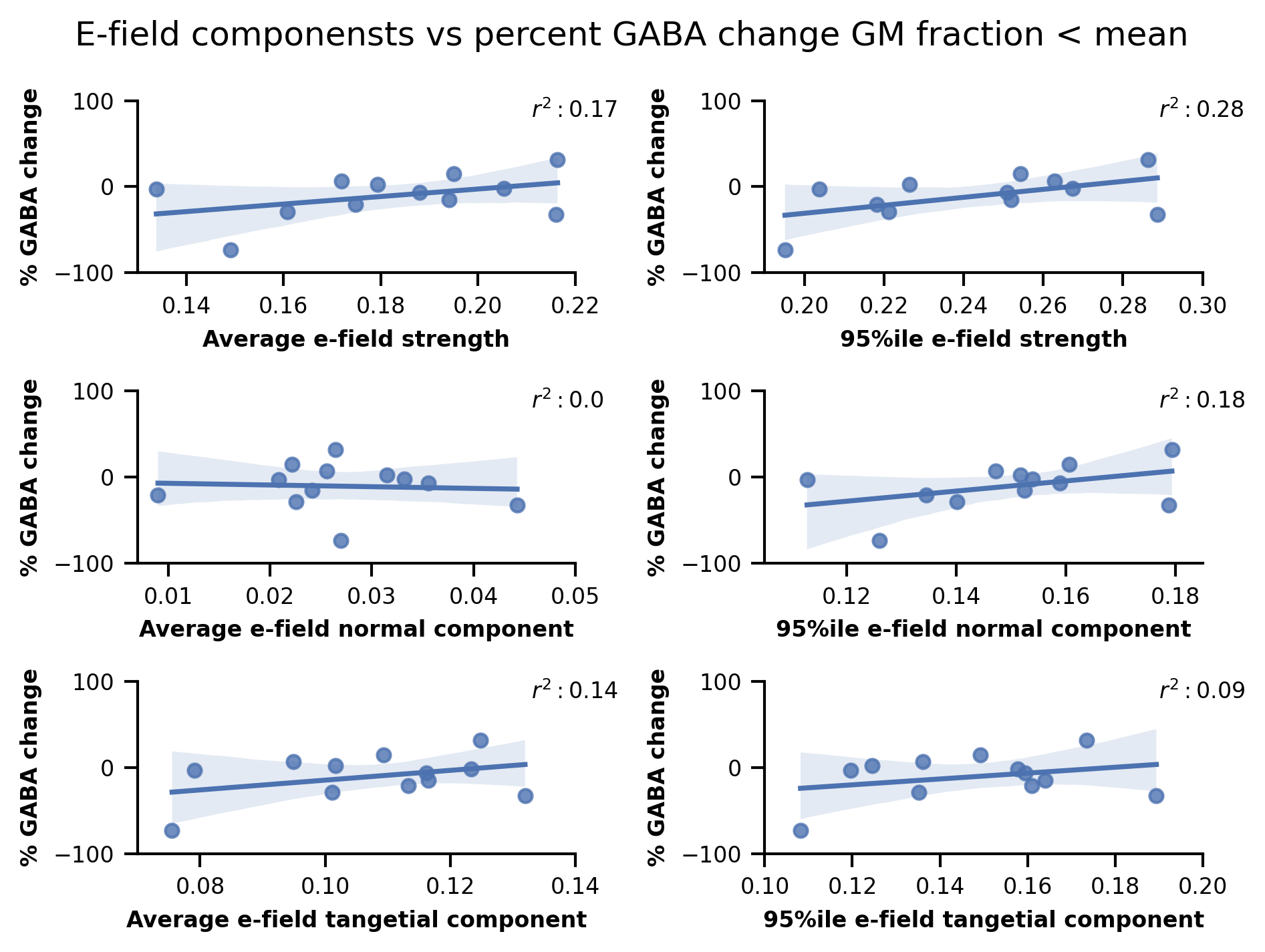
**

*Supplementary figure 3: Association between all the E-field estimates (magnitude, normal and tangent; mean and 95th percentile), in the M1 MRS voxel, and the percent GABA change, only for participants with grey matter partial volume estimates lower than the mean.*

*
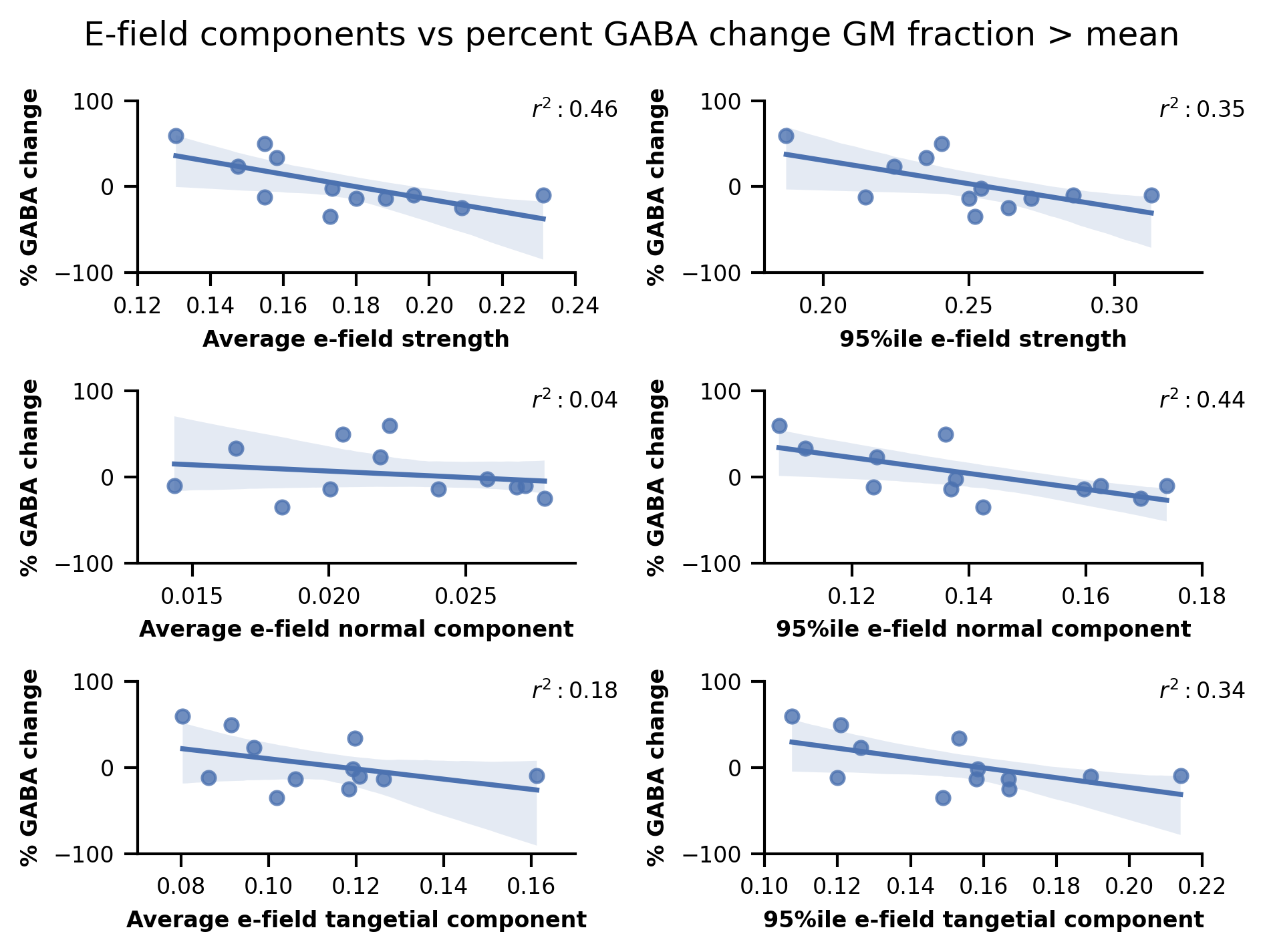
*

*Supplementary figure 4: Association between all the E-field estimates (magnitude, normal and tangent; mean and 95th percentile), in the M1 MRS voxel, and the percent GABA change, only for participants with grey matter partial volume estimates greater than the mean.*

*
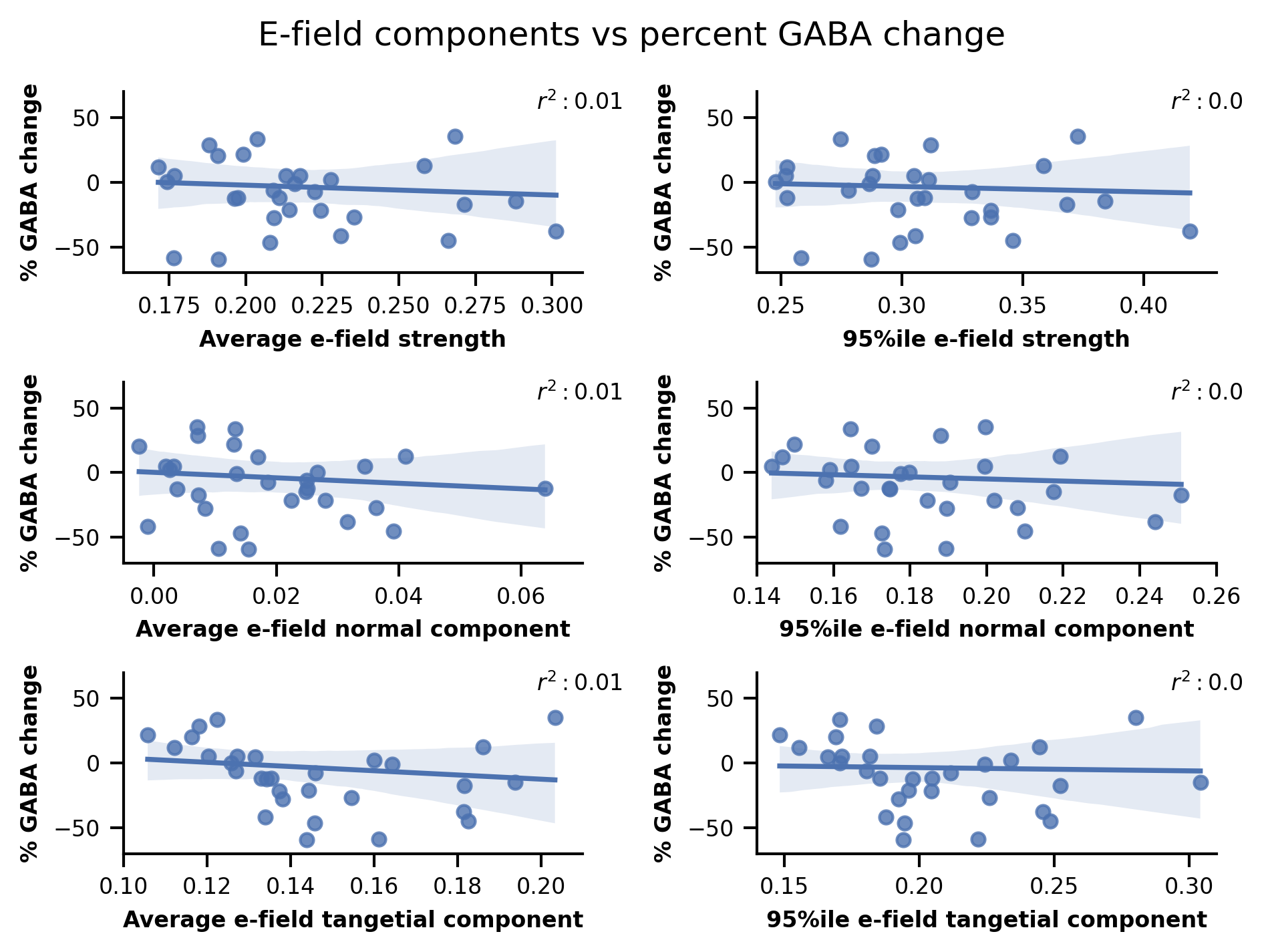
*

*Supplementary figure 5: Association between all the E-field estimates (magnitude, normal and tangent; mean and 95th percentile), in the temporal MRS voxel, and the percent GABA change.*

*
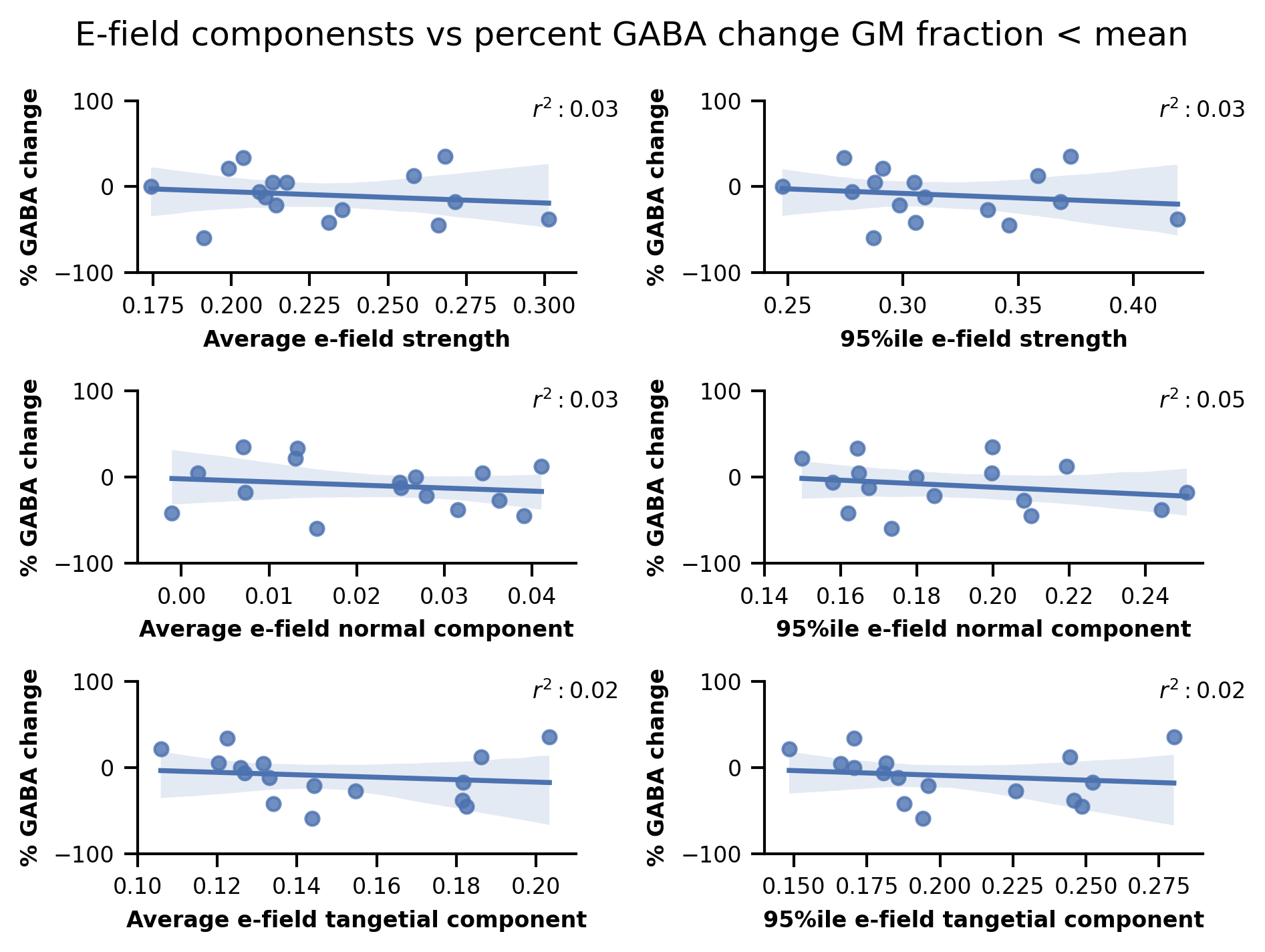
*

*Supplementary figure 6: Association between all the E-field estimates (magnitude, normal and tangent; mean and 95th percentile), in the temporal MRS voxel, and the percent GABA change, only for participants with grey matter partial volume estimates lower than the mean.*

*
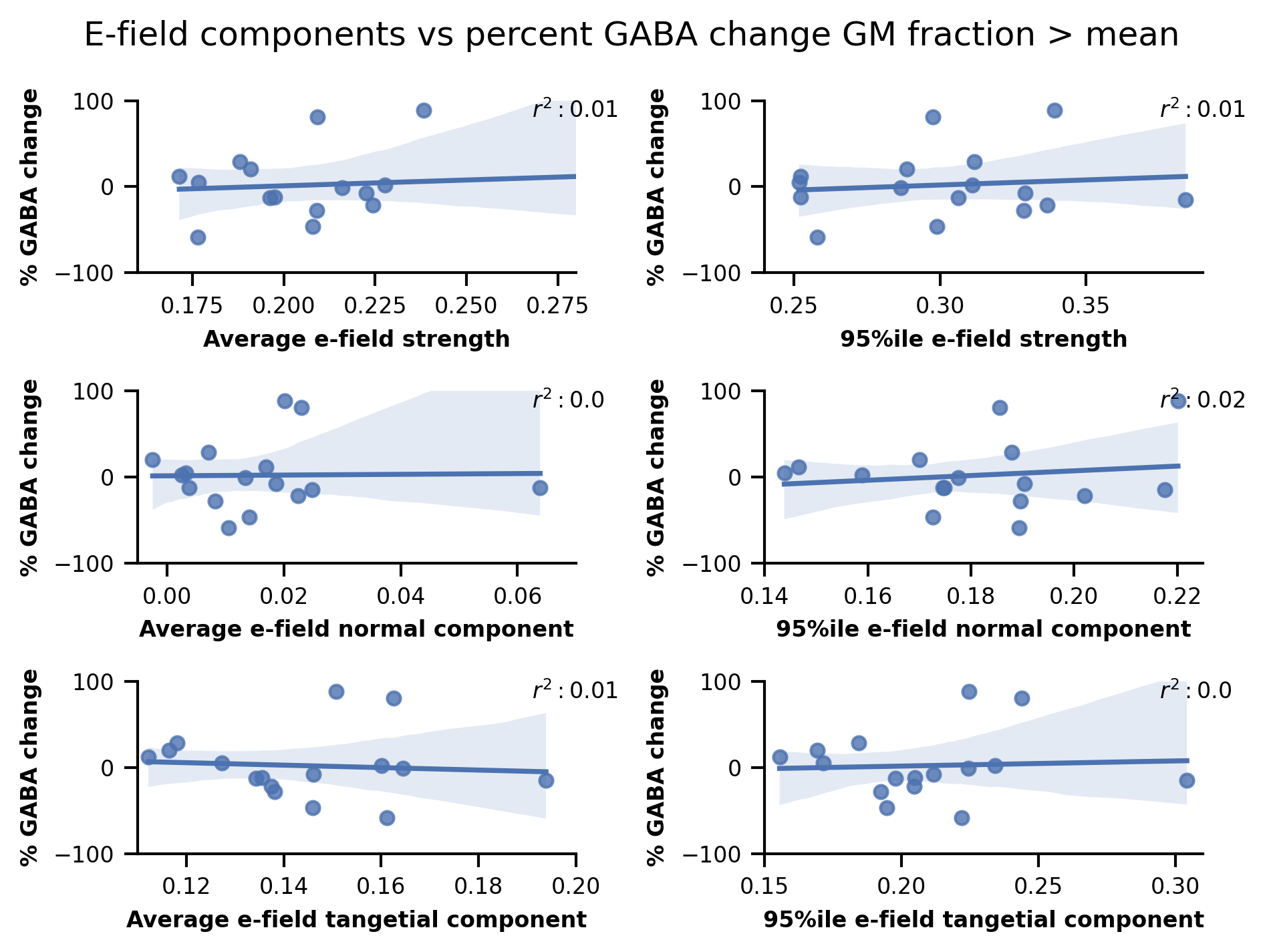
*

*Supplementary figure 7: Association between all the E-field estimates (magnitude, normal and tangent; mean and 95th percentile), in the temporal MRS voxel, and the percent GABA change, only for participants with grey matter partial volume estimates greater than the mean.*


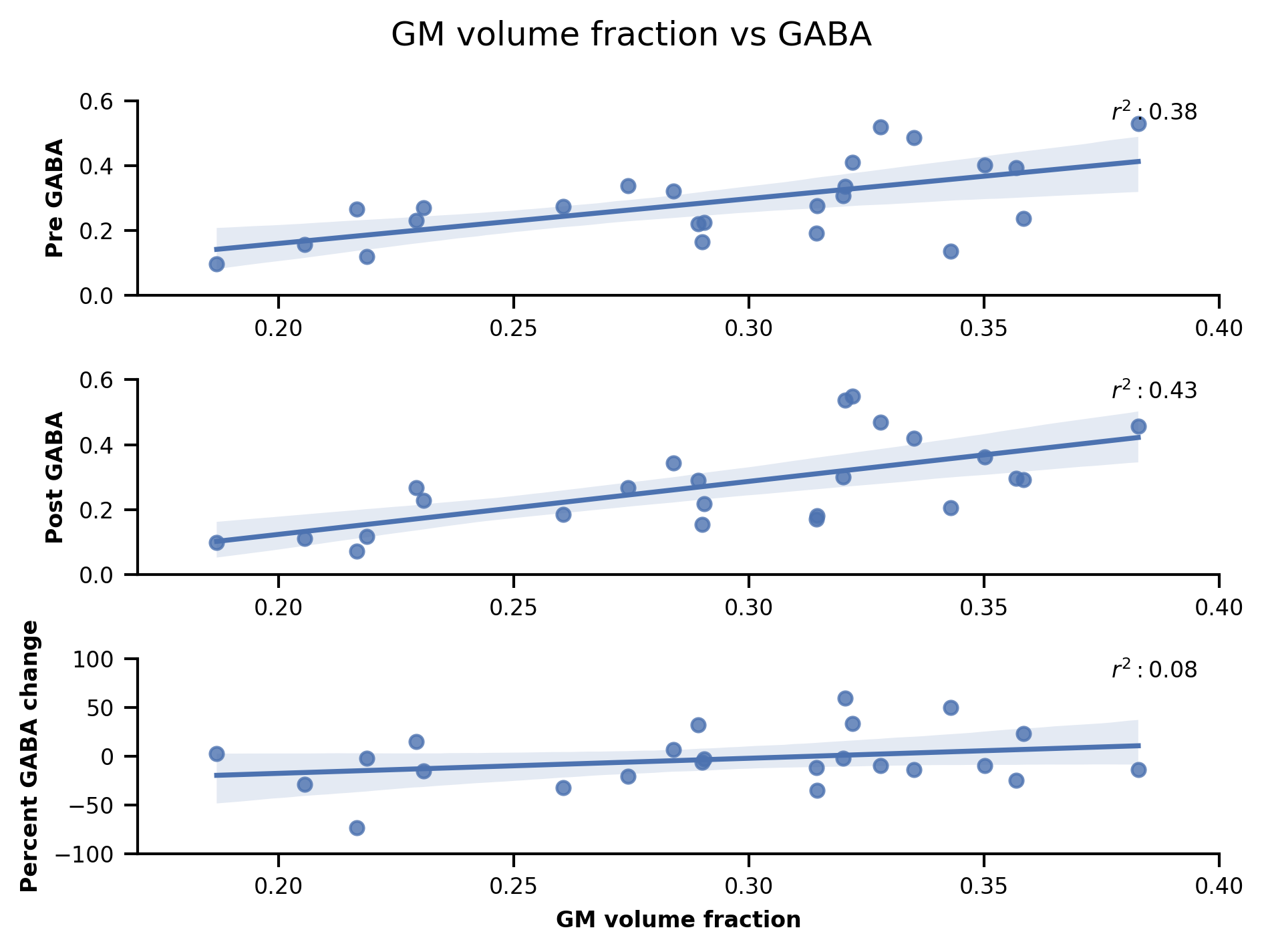

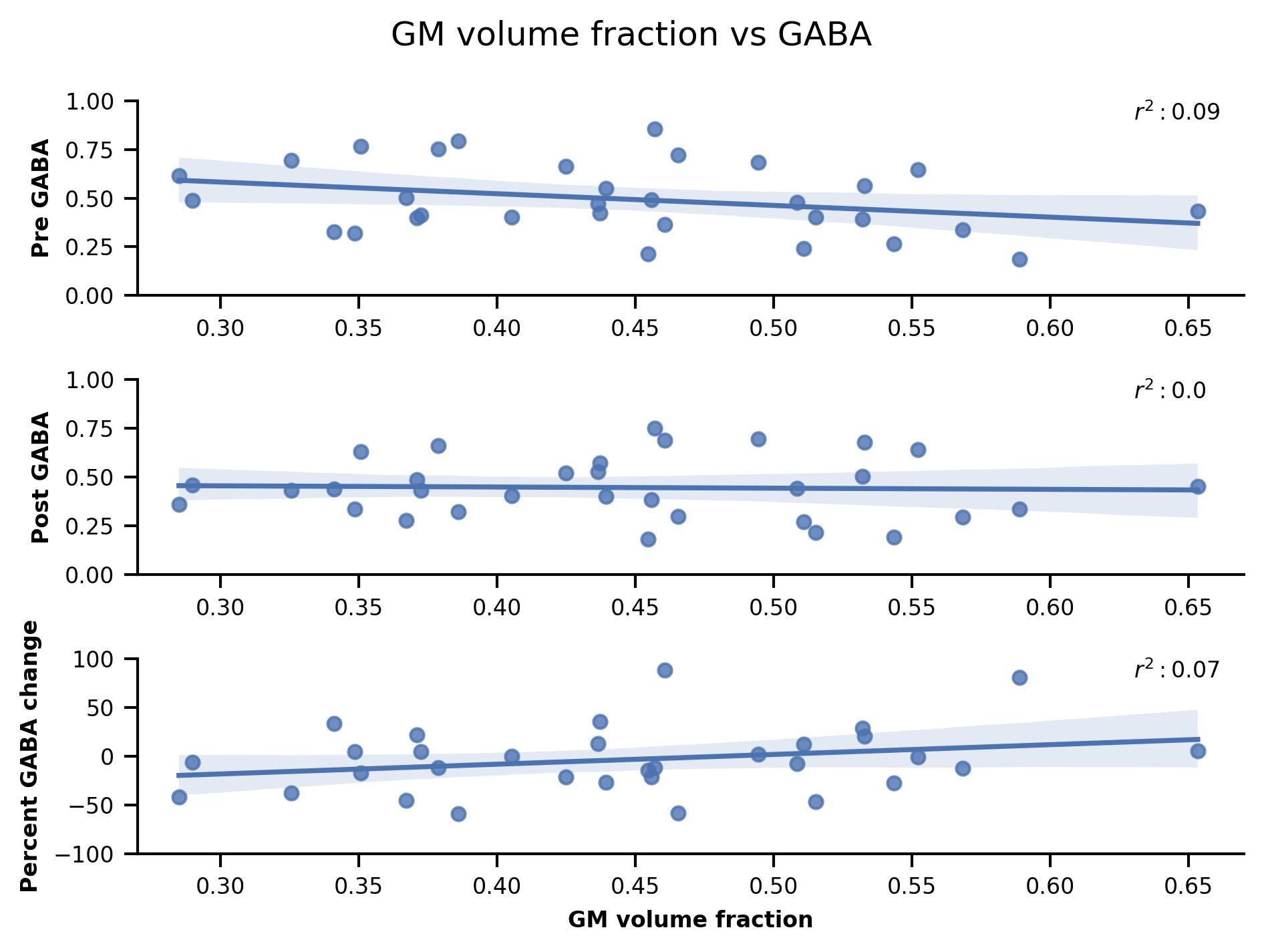
*Supplementary figure 8: Gaba (ratio to creatine) vs GM volume fraction in M1 (top) and temporal (bottom) area*


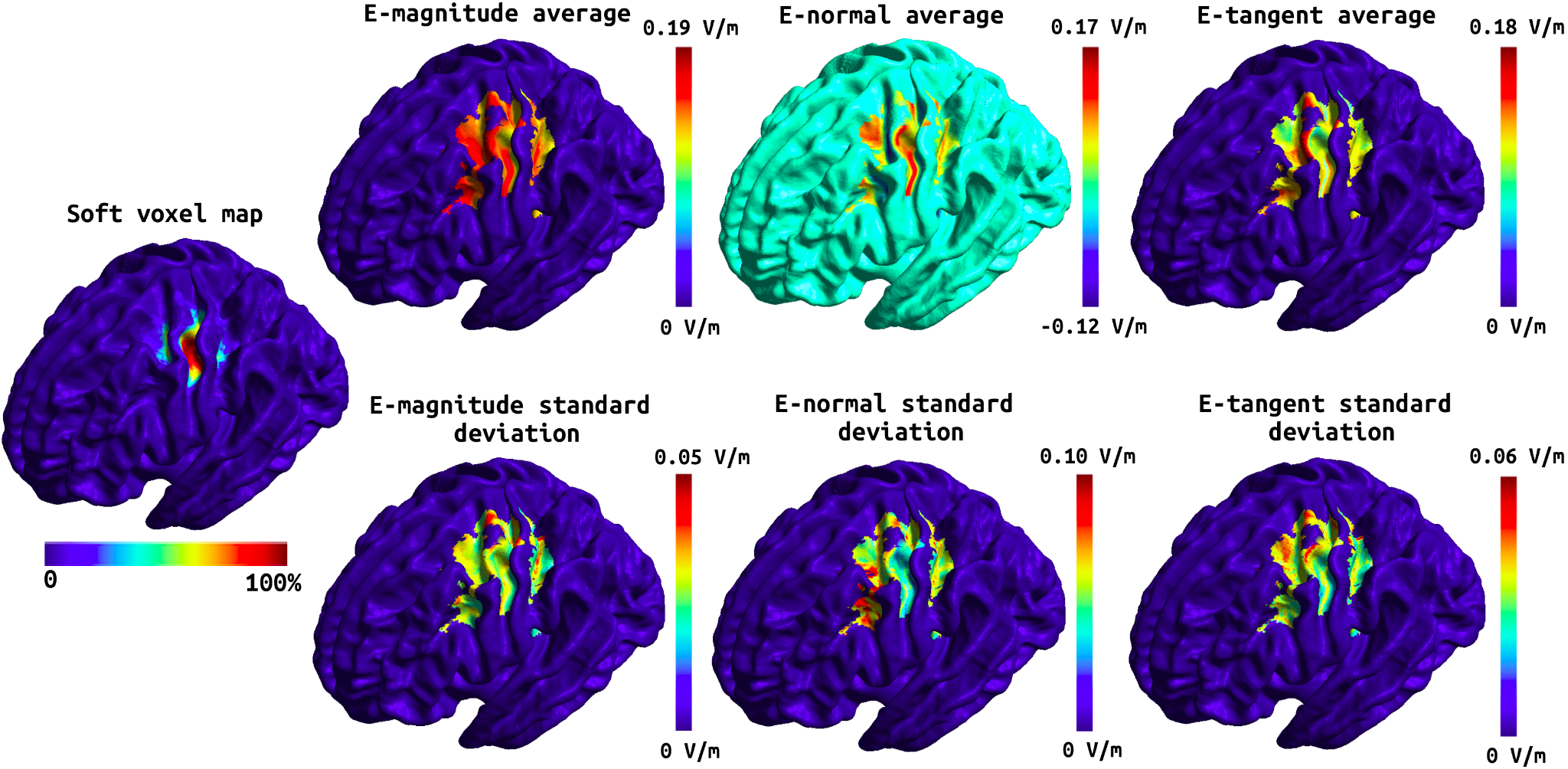
*Supplementary figure 9: Soft voxel map (leftmost) and the mean and standard deviation of the E-field components over the subjects in fsaverage space for the tDCS stimulation targeting the M1. First column: the mean E-field magnitude (top) and its standard deviation (bottom). Second column: the mean E-field normal component (top) and its standard deviation (bottom). Third column: the mean E-field tangential component (top) and its standard deviation (bottom).*

*
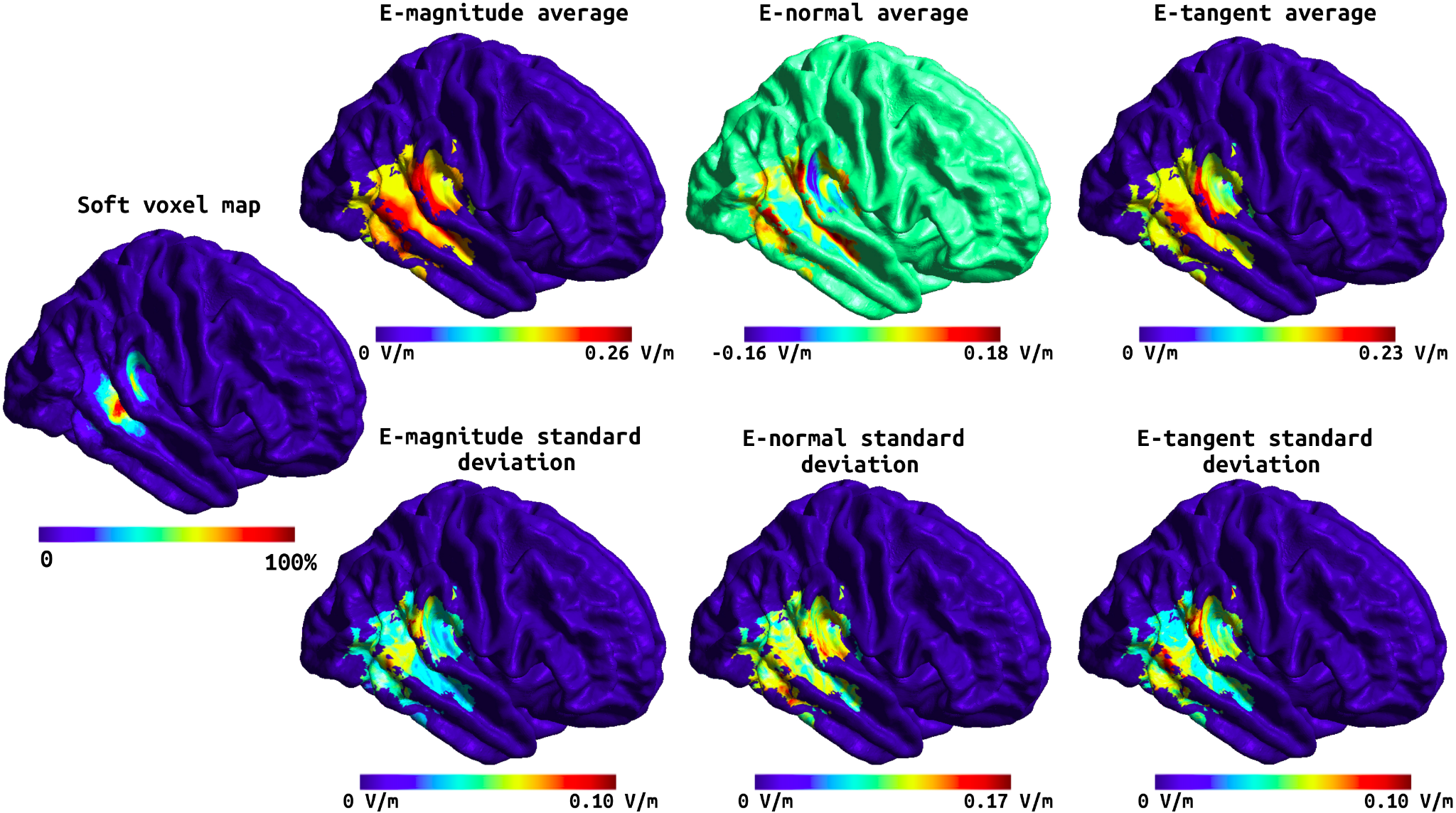
*

*Supplementary figure 10: Soft voxel map (leftmost) and the mean and standard deviation of the E-field components over the subjects in fsaverage space for the tDCS stimulation targeting the temporal cortex. First column: the mean E-field magnitude (top) and its standard deviation (bottom). Second column: the mean E-field normal component (top) and its standard deviation (bottom). Third column: the mean E-field tangential component (top) and its standard deviation (bottom).*

*Supplementary table 1: GABA data quality (included participants only)*

| ***Dataset*** | ***SNR*** | ***CRLB*** | ***NAA fwhm*** | ***Model fwhm*** | ***Creatine fwhm*** |
| --- | --- | --- | --- | --- | --- |
| ***Dataset 1 (unpublished)*** | *50.5 ± 5.8* | *21.3 ± 4.8* |  | *9.6 ± 2.5* |  |
| ***Koolschijn et al. 2019*** | *46.2 ± 9.6* | *18.6 ± 7.3* |  | *10.9 ± 1.4* |  |
| ***Bachtiar et al. 2018*** | *46.8 ± 6.8* | *20.1 ± 7.2* |  | *9.9 ± 2.0* |  |
| ***Barron et al. 2016*** | *51.2 ± 6.8* | *20.0 ± 6.4* |  | *9.9 ± 1.3* |  |
| ***Bachtiar et al. 2015*** |  |  | *9.1 ± 0.6* |  | *8.3 ± 0.3* |

*SNR - signal to noise ratio, CRLB -* Cramér-Rao lower bounds, fwhm - full width at half maximum

SNR, CRLB and NAA fwhm were used for quality control during the analysis. We also provide the Model fwhm, estimated using LCModel for Semi-LASER data and Creatine fwhm, estimated using Gannet for MEGA-PRESS data since these are approximately comparable.

*Supplementary table 2: M1 model results*

| **E-field variable** | **Main effects** | | | **Two-way interactions** | | | **Three-way interaction** |
| --- | --- | --- | --- | --- | --- | --- | --- |
|  | **Time** | **Efield** | **Grey matter volume** | **Time*Efield** | **Time*Grey matter volume** | ***Efield*Grey matter volume*** | ***Time*Efield*Grey matter volume*** |
| **Mean: magnitude** | t(24) = -3.35  **p=0.003*** | t(30.27) = -1.89  p = 0.07 | t(30.27) = -1.66  p = 0.107 | t(24) = 3.24  **p = 0.003*** | t(24) = 3.64  **p = 0.001*** | *t(30.27) = 2.16*  ***p = 0.039**** | *t(24) = -3.55*  ***p = 0.002**** |
| **95^th^ percentile: magnitude** | t(24)= -3.31  **p=0.003*** | t(30.62) = -1.77  p = 0.086 | t(30.62) = -1.62  p = 0.115 | t(24) = 3.18  **p = 0.004*** | t(24) = 3.53  **p = 0.002*** | t(24) = 2.01  p = 0.052 | *t(24) = -3.41*  ***p = 0.002**** |
| **Mean: normal component** | t(24) = -0.37  p = 0.713 | t(33.66) = -0.40  p= 0.69 | t(33.66) = 0.39  p= 0.698 | t(24) = 0.22  p = 0.824 | t(24) = 0.42  p = 0.677 | t(33.66) = 0.30  p = 0.767 | t(24) = -0.31  p = 0.763 |
| **95^th^ percentile: normal component** | t(24) = -3.46  **p=0.002*** | t(29.56) = -1.40  p = 0.172 | t(29.56) = -1.14  p = 0.263 | t(24) = 3.38  **p = 0.002*** | t(24) = 3.73  **p = 0.001*** | t(29.56) = 1.53  p = 0.137 | *t(24) = -3.67*  ***p = 0.001**** |
| **Mean: tangential component** | t(24) = -2.82  **p=0.010*** | t(33.05) = -2.34  **p = 0.026*** | t(33.05) = -2.12  **p = 0.042*** | t(24) = 2.66  **p = 0.014*** | t(24) = 2.96  **p = 0.007*** | t(33.05) = 2.72  **p = 0.010*** | t(24) = -2.82  **p = 0.009*** |
| **95^th^ percentile: tangential component** | t(24) = -2.37  **p=0.026*** | t(32.48) = -1.70  p = 0.100 | t(32.48) = -1.53  p = 0.136 | t(24) = 2.17  **p = 0.041*** | t(24) = 2.66  **p = 0.014*** | t(32.48) = 2.13  **p = 0.041*** | t(24) = -2.47  **p = 0.021*** |

*Supplementary table 3: M1 posthoc tests for significant three-way interactions*

| **Efield variable** | **25^th^ percentile**  **Grey matter volume** | **75^th^ percentile**  **Grey matter volume** |
| --- | --- | --- |
| **Mean: magnitude** | Chisq(1) = 1.78  p = 0.182 | Chisq(1) = 12.91  **p < 0.001*** |
| **95^th^ percentile: magnitude** | Chisq(1) = 1.93  p = 0.165 | Chisq(1) = 9.72  **p = 0.002*** |
| **Mean: normal component** | - | - |
| **95^th^ percentile: normal component** | Chisq(1) = 2.95  p = 0.086 | Chisq(1) = 14.69  **p < 0.001*** |
| **Mean: tangential component** | Chisq(1) = 2.00  p = 0.157 | Chisq(1) = 5.31  **p = 0.021*** |
| **95^th^ percentile: tangential component** | Chisq(1) = 0.31  p = 0.581 | Chisq(1) = 7.57  **p = 0.006*** |

*Supplementary table 4: Temporal model results*

| **Efield variable** | **Main effects** | | | **Two-way interactions** | | | **Three-way interaction** |
| --- | --- | --- | --- | --- | --- | --- | --- |
|  | **Time** | **Efield** | **Grey matter volume** | **Time*Efield** | **Time*Grey matter volume** | ***Efield*Grey matter volume*** | ***Time*Efield*Grey matter volume*** |
| **Mean: magnitude** | t(28) = 0.87  p= 0.393 | t(44.87) = 1.67  p = 0.102 | t(44.87) = 1.40  p = 0.170 | t(28) = -1.04  p = 0.310 | t(28) = -1.03  p = 0.314 | *t(44.87) = -1.53*  *p = 0.133* | *t(28) = 1.16*  *p = 0.255* |
| **95^th^ percentile: magnitude** | t(28) = 1.54  p= 0.135 | t(43.64) = 2.06  **p = 0.045*** | t(43.64) = 1.82  p = 0.076 | t(28) = -1.71  p = 0.098 | t(28) = -1.79  p = 0.085 | *t(43.64) = -1.97*  *p = 0.055* | *t(28) = 1.92*  *p = 0.065* |
| **Mean: normal component** | t(28) = 0.54  p= 0.591 | t(44.90) = 0.36  p = 0.717 | t(44.90) = -0.59  p = 0.557 | t(28) = -1.22  p = 0.231 | t(28) = -0.47  p = 0.639 | *t(44.90) = -0.61*  *p = 0.523* | *t(28) = 1.08*  *p = 0.291* |
| **95^th^ percentile: normal component** | t(28) = 1.34  p= 0.193 | t(44.17) = 1.70  p = 0.097 | t(44.17) = 1.34  p = 0.189 | t(28) = -1.48  p = 0.151 | t(28) = -1.32  p = 0.198 | *t(44.17) = -1.49*  *p = 0.144* | *t(28) = 1.43*  *p = 0.164* |
| **Mean: tangential component** | t(28) = 0.59  p= 0.564 | t(46.27) = 1.95  p = 0.057 | t(46.27) = 1.49  p = 0.144 | t(28) = -0.71  p = 0.483 | t(28) = -0.50  p = 0.618 | *t(*46.27*) = -1.69*  *p = 0.099* | *t(28) = 0.62*  *p = 0.542* |
| **95^th^ percentile: tangential component** | t(28) = 0.68  p= 0.501 | t(46.02) = 2.15  **p = 0.037*** | t(46.02) = 1.77  p = 0.084 | t(28) = -0.82  p = 0.422 | t(28) = -0.64  p = 0.530 | *t(*46.02*) = -1.99*  *p = 0.053* | *t(28) = 0.76*  *p = 0.454* |


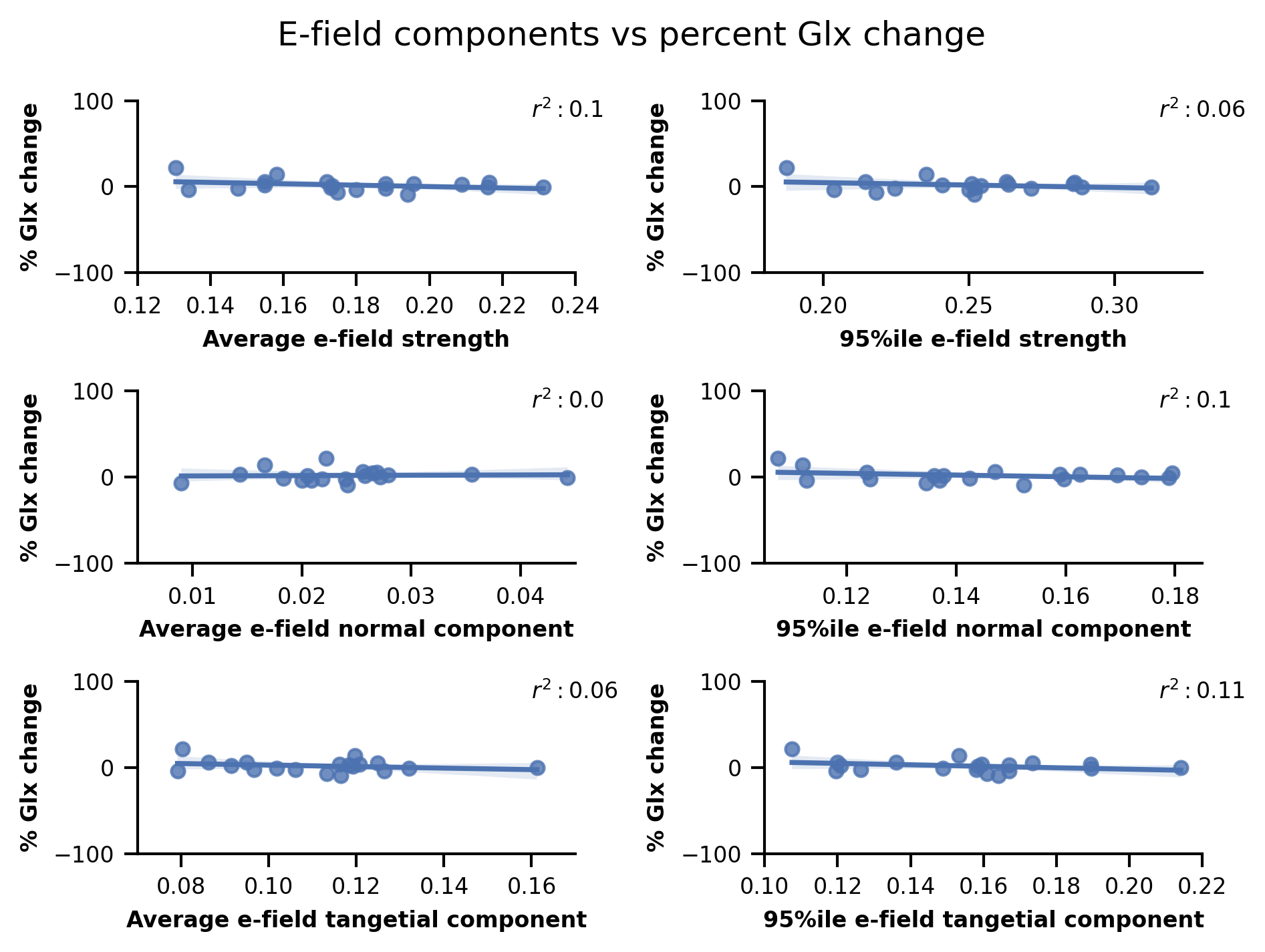
*Supplementary figure 11: Association between all the E-field estimates (magnitude, normal and tangent; mean and 95th percentile), in the M1 MRS voxel, and the percent Glx (glutamate + glutamine) change.*


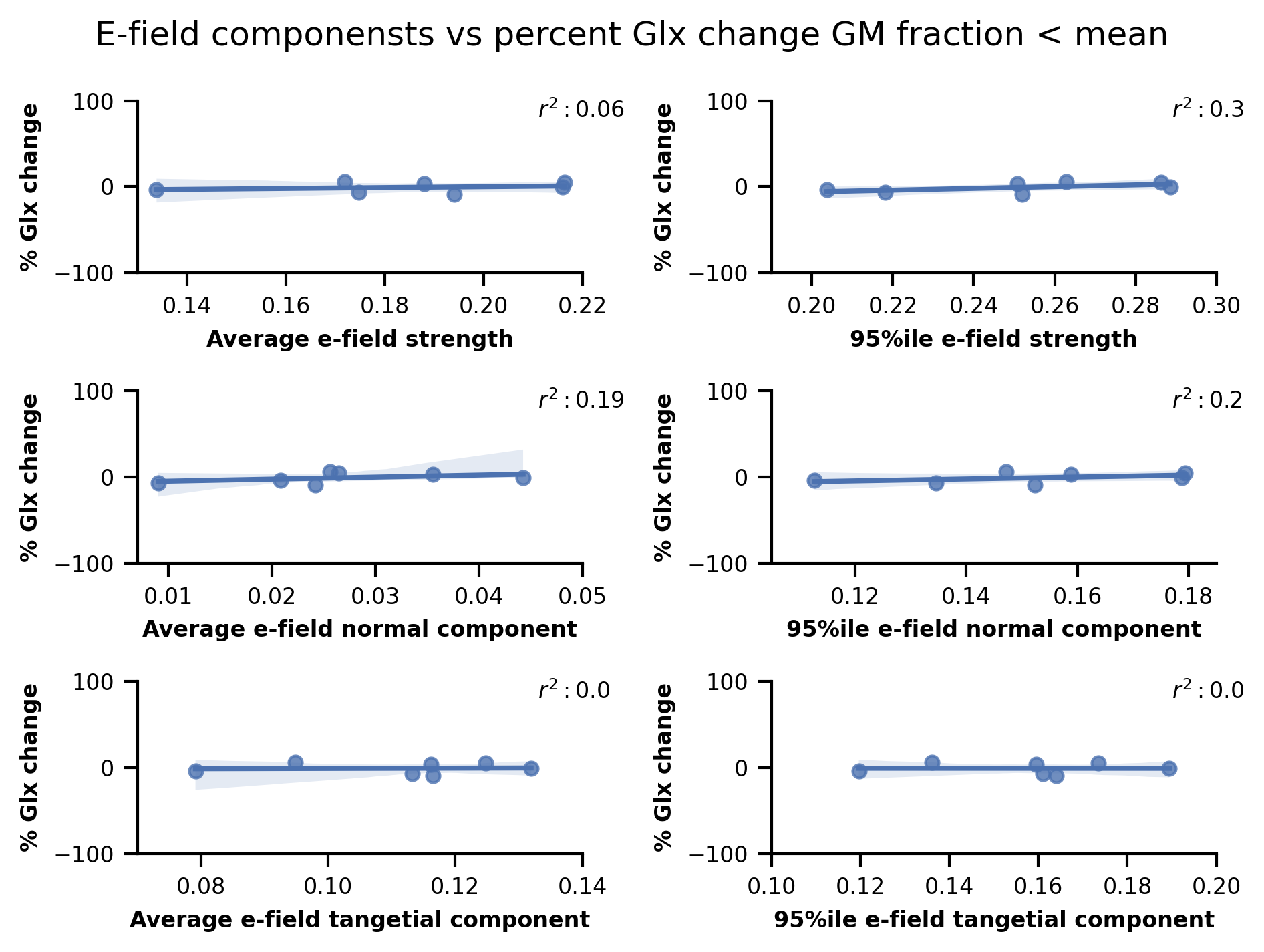
*Supplementary figure 12: Association between all the E-field estimates (magnitude, normal and tangent; mean and 95th percentile), in the M1 MRS voxel, and the percent Glx (glutamate + glutamine) change, only for participants with grey matter partial volume estimates lower than the mean.*


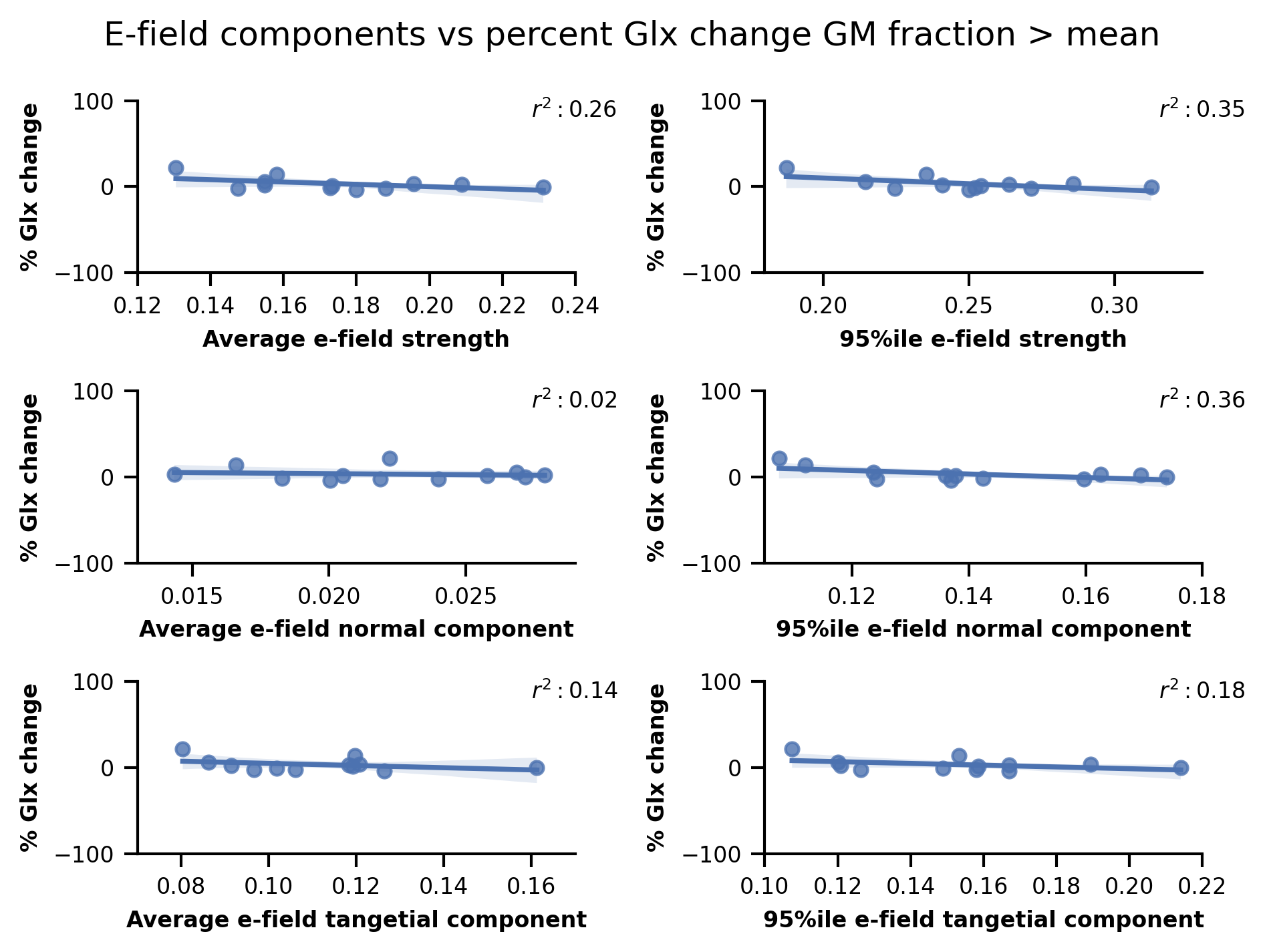
*Supplementary figure 13: Association between all the E-field estimates (magnitude, normal and tangent; mean and 95th percentile), in the M1 MRS voxel, and the percent Glx (glutamate + glutamine) change, only for participants with grey matter partial volume estimates greater than the mean.*


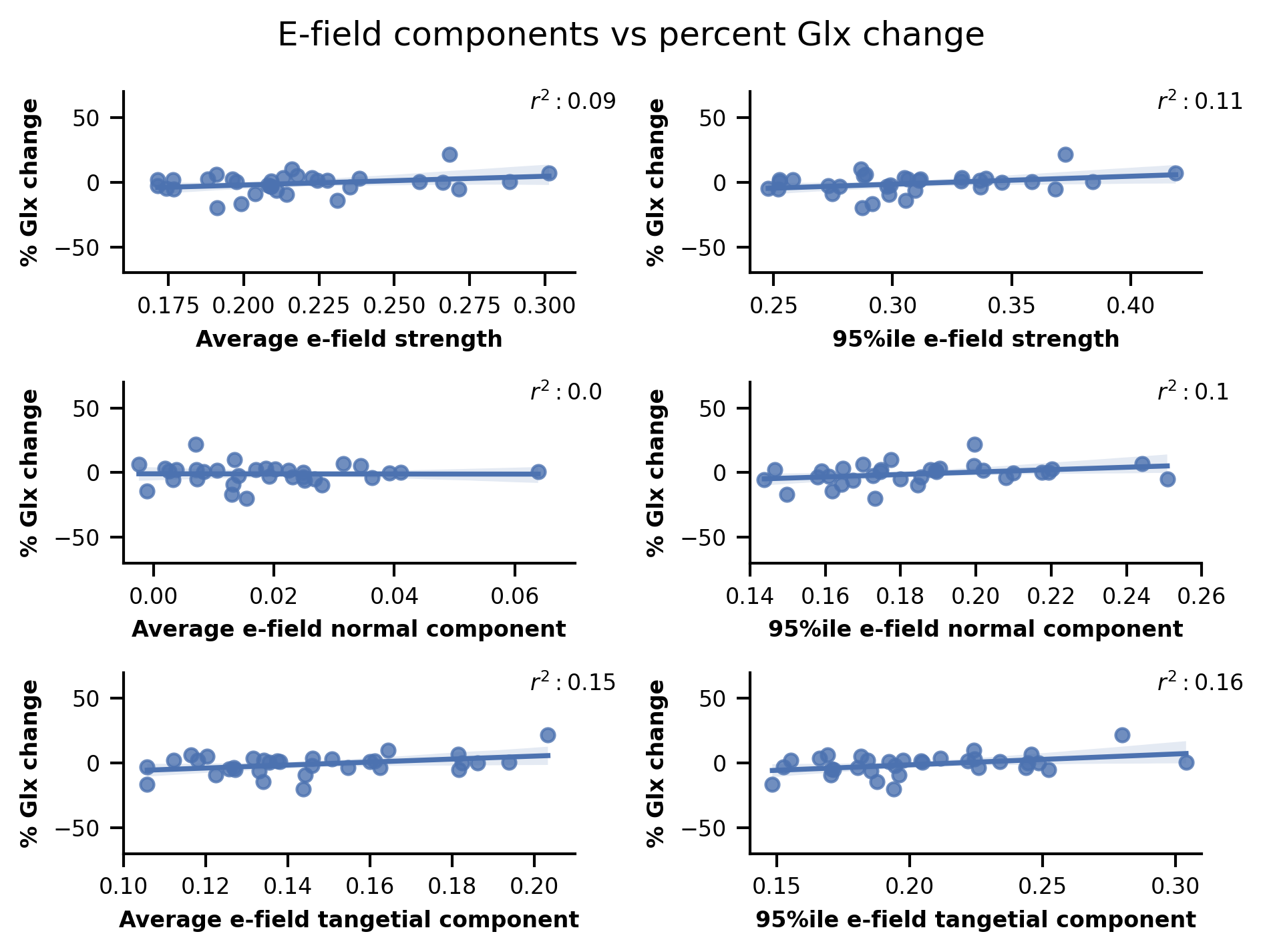
*Supplementary figure 14: Association between all the E-field estimates (magnitude, normal and tangent; mean and 95th percentile), in the temporal MRS voxel, and the percent Glx (glutamate + glutamine) change.*


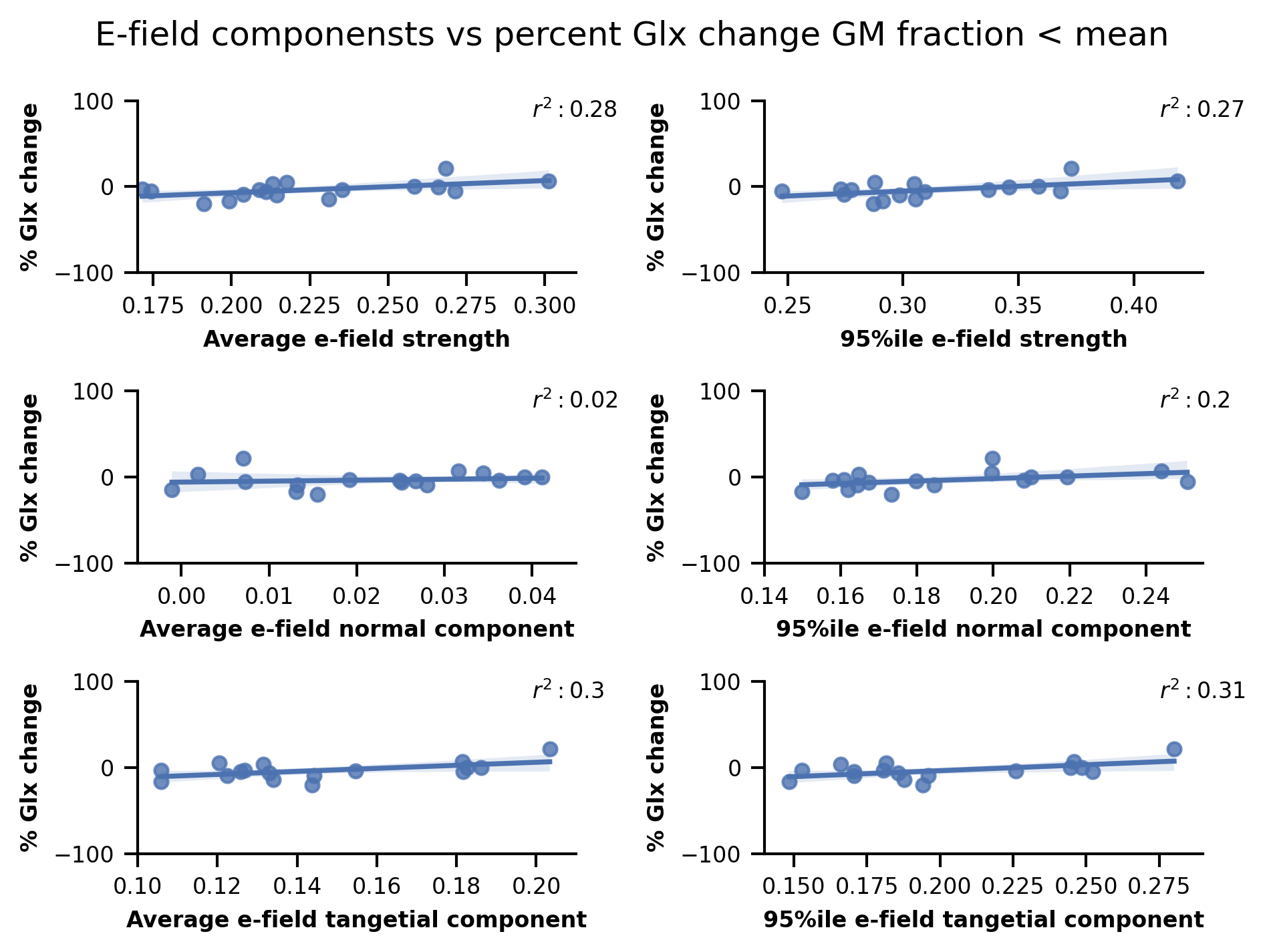
*Supplementary figure 15: Association between all the E-field estimates (magnitude, normal and tangent; mean and 95th percentile), in the temporal MRS voxel, and the percent Glx (glutamate + glutamine) change, only for participants with grey matter partial volume estimates lower than the mean.*


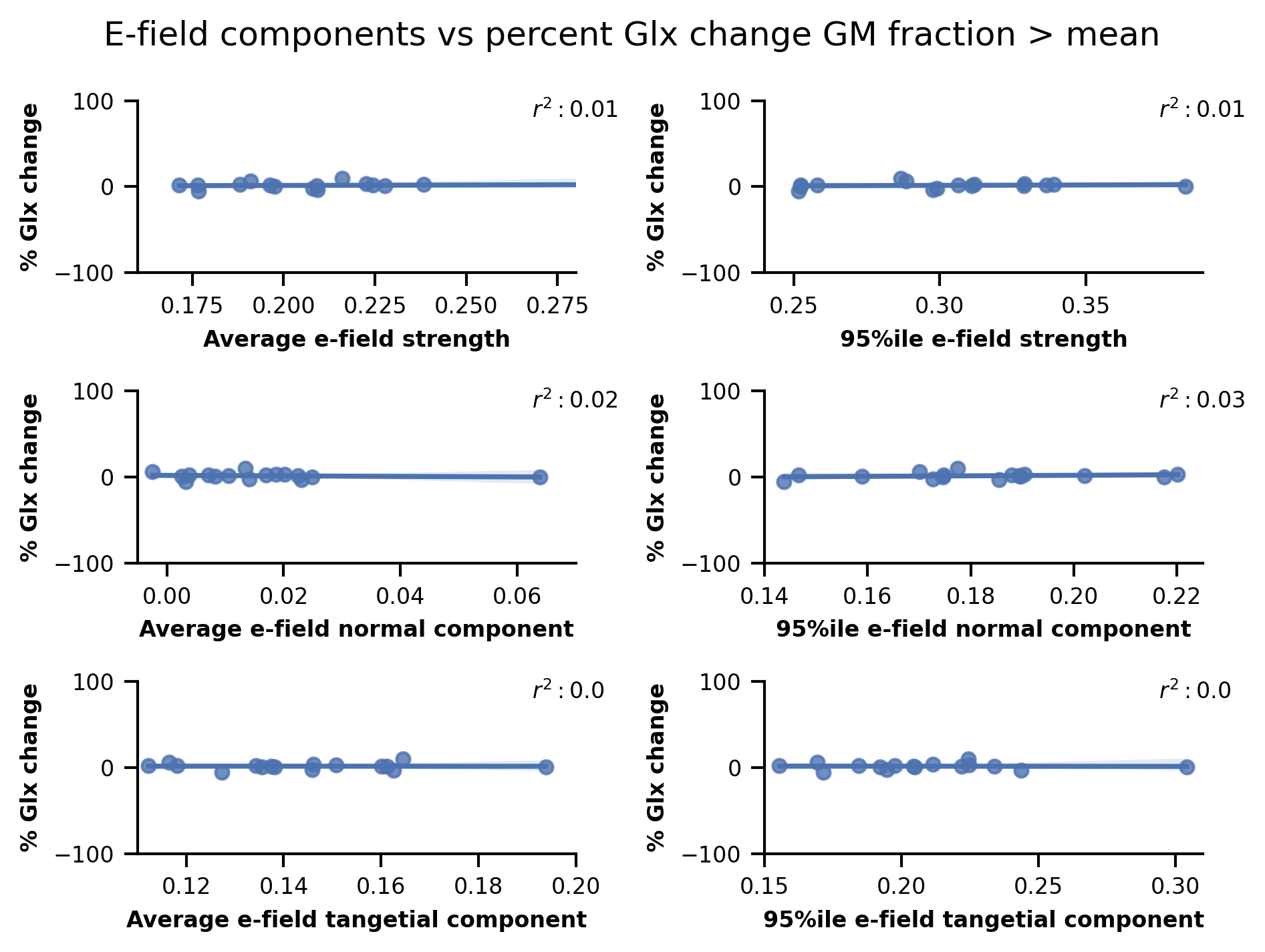


*Supplementary figure 16: Association between all the E-field estimates (magnitude, normal and tangent; mean and 95th percentile), in the temporal MRS voxel, and the percent Glx (glutamate + glutamine) change, only for participants with grey matter partial volume estimates greater than the mean.*


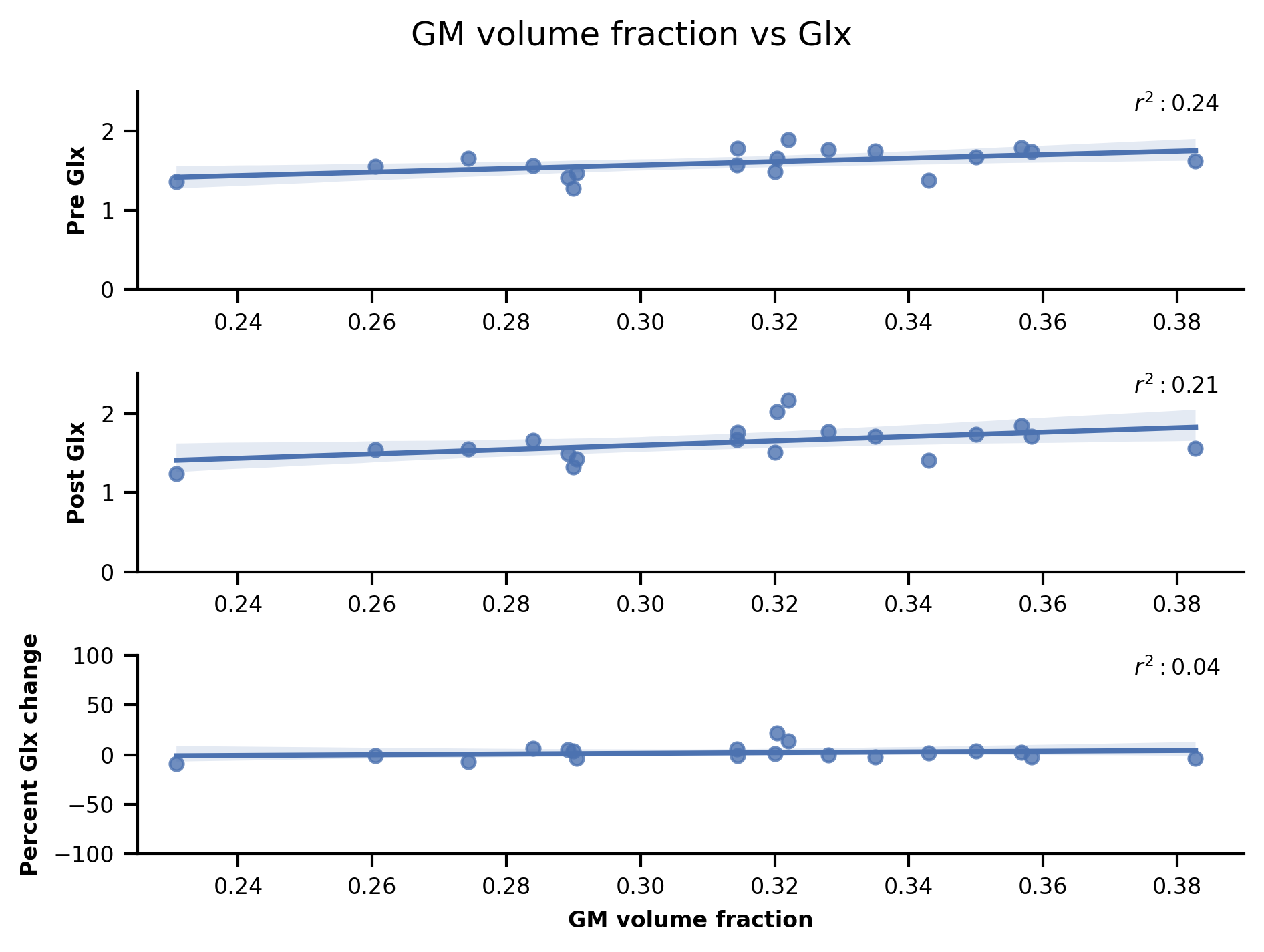


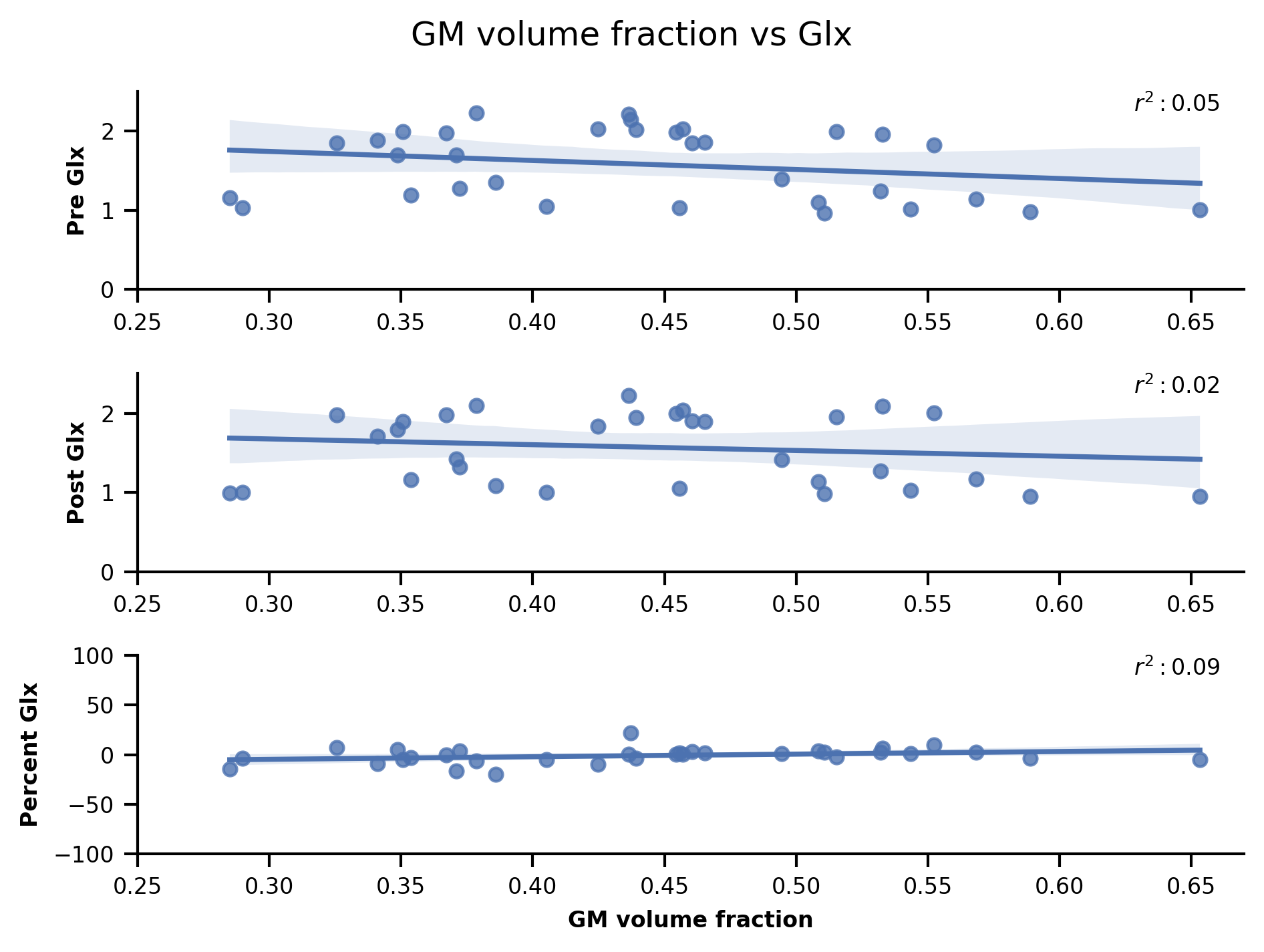


*Supplementary figure 17: Glx (glutamate + glutamine; ratio to creatine) vs GM volume fraction in M1 (top) and temporal (bottom) area*

*Supplementary table 5: M1 model results for Glx (glutamate + glutamine)*

| **E-field variable** | **Main effects** | | | **Two-way interactions** | | | **Three-way interaction** |
| --- | --- | --- | --- | --- | --- | --- | --- |
|  | **Time** | **Efield** | **Grey matter volume** | **Time*Efield** | **Time*Grey matter volume** | ***Efield*Grey matter volume*** | ***Time*Efield*Grey matter volume*** |
| **Mean: magnitude** | t(19) = 0.03  p=0.977 | t(23.2) = -0.649  p = 0.523 | t(23.2) = -0.444  p = 0.661 | t(19) = ~~-~~0.084  p = 0.934 | t(19) = 0.104  p = 0.918 | t(23.2) = 0.677  p = 0.505 | t(19) = -0.039  p = 0.969 |
| **95^th^ percentile: magnitude** | t(19)= -1.100  p=0.286 | t(22.9) = -0.455  p = 0.654 | t(22.9) = -0.295  p = 0.770 | t(19) = 1.042  p = 0.310 | t(19) = 1.217  p = 0.239 | t(22.9) = 0.471  p = 0.642 | t(19) = -1.150  p = 0.264 |
| **Mean: normal component** | t(19) = -0.412  p = 0.685 | t(23.7) = -0.126  p= 0.901 | t(23.7) = 0.437  p= 0.666 | t(19) = 0.228  p = 0.822 | t(19) = 0.409  p = 0.687 | t(23.7) = 0.018  p = 0.986 | t(19) = -0.197  p = 0.846 |
| **95^th^ percentile: normal component** | t(19) = -0.351  p=0.730 | t(23.2) = -0.724  p = 0.476 | t(23.2) = -0.466  p = 0.645 | t(19) = 0.301  p = 0.767 | t(19) = 0.501  p = 0.622 | t(23.2) = 0.701  p = 0.490 | t(19) = -0.440  p = 0.665 |
| **Mean: tangential component** | t(19) = -0.309  p=0.761 | t(23.2) = -0.111  p = 0.912 | t(23.2) = -0.064  p = 0.949 | t(19) = 0.236  p = 0.816 | t(19) = 0.405  p = 0.690 | t(23.2) = 0.159  p = 0.875 | t(19) = -0.320  p = 0.753 |
| **95^th^ percentile: tangential component** | t(19) = 0.334  p=0.742 | t(23.1) = -0.133  p = 0.895 | t(23.1) = 0.051  p = 0.960 | t(19) = -0.407  p = 0.689 | t(19) = -0.205  p = 0.840 | t(23.1) = 0.192  p = 0.850 | t(19) = 0.290  p = 0.775 |

*Supplementary table 6: Temporal model results for Glx (glutamate + glutamine)*

| **E-field variable** | **Main effects** | | | **Two-way interactions** | | | **Three-way interaction** |
| --- | --- | --- | --- | --- | --- | --- | --- |
|  | **Time** | **Efield** | **Grey matter volume** | **Time*Efield** | **Time*Grey matter volume** | ***Efield*Grey matter volume*** | ***Time*Efield*Grey matter volume*** |
| **Mean: magnitude** | t(33) = -0.649  p=0.521 | t(34.6) = -0.321  p = 0.750 | t(34.6) = -0.941  p = 0.353 | t(33) = 0.247  p = 0.806 | t(33) = 0.104  p = 0.918 | t(34.6) = 0.875  p = 0.387 | t(33) = 0.254  p = 0.801 |
| **95^th^ percentile: magnitude** | t(33)= -0.786  p=0.438 | t(34.4) = 0.753  p = 0.457 | t(34.4) = 0.225  p = 0.823 | t(33) = 0.473  p = 0.639 | t(33) = 0.283  p = 0.779 | t(34.4) = -0.355  p = 0.725 | t(33) = -0.006  p = 0.995 |
| **Mean: normal component** | t(33) = -1.843  p = 0.074 | t(34.5) = 0.465  p= 0.645 | t(34.5) = -0.466  p= 0.644 | t(33) = 1.043  p = 0.304 | t(33) = 1.866  p = 0.071 | t(34.5) = -0.180  p = 0.858 | t(33) = -1.045  p = 0.304 |
| **95^th^ percentile: normal component** | t(33) = -0.752  p=0.458 | t(34.5) = 0.582  p = 0.564 | t(34.5) = -0.101  p = 0.920 | t(33) = 0.390  p = 0.699 | t(33) = 0.241  p = 0.811 | t(34.5) = -0.042  p = 0.967 | t(33) = 0.062  p = 0.951 |
| **Mean: tangential component** | t(33) = -0.927  p=0.361 | t(34.6) = 0.857  p = 0.398 | t(34.6) = 0.089  p = 0.930 | t(33) = 0.627  p = 0.535 | t(33) = 0.437  p = 0.665 | t(34.6) = -0.319  p = 0.752 | t(33) = -0.177  p = 0.860 |
| **95^th^ percentile: tangential component** | t(33) = -1.258  p=0.217 | t(34.6) = 1.491  p = 0.145 | t(34.6) = 0.730  p = 0.470 | t(33) = 0.981  p = 0.334 | t(33) = 0.788  p = 0.436 | t(34.6) = -1.002  p = 0.323 | t(33) = -0.549  p = 0.587 |


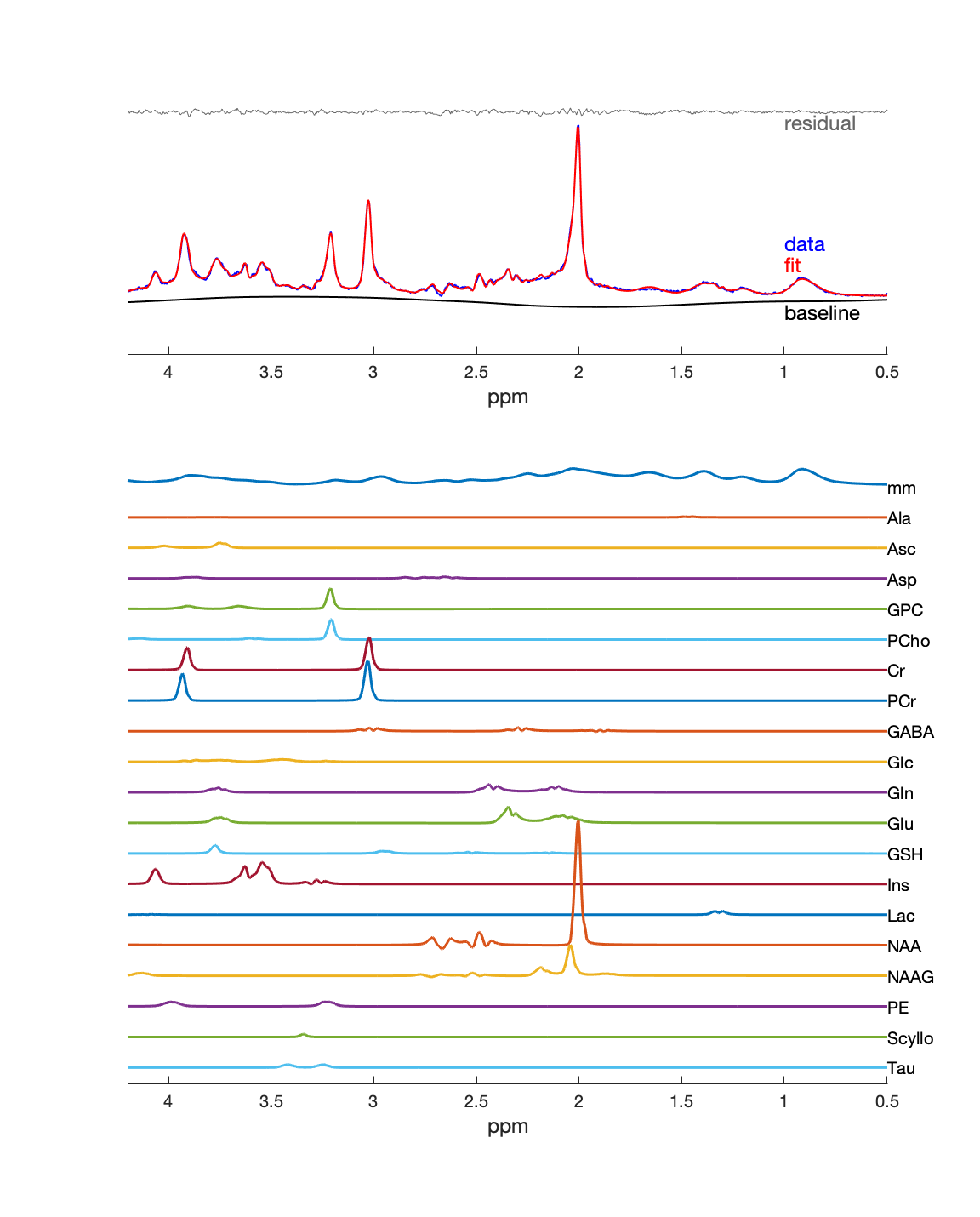


Supplementary figure 18: Example SemiLASER spectrum showing raw data, model fit, residual noise and individual metabolite fits.


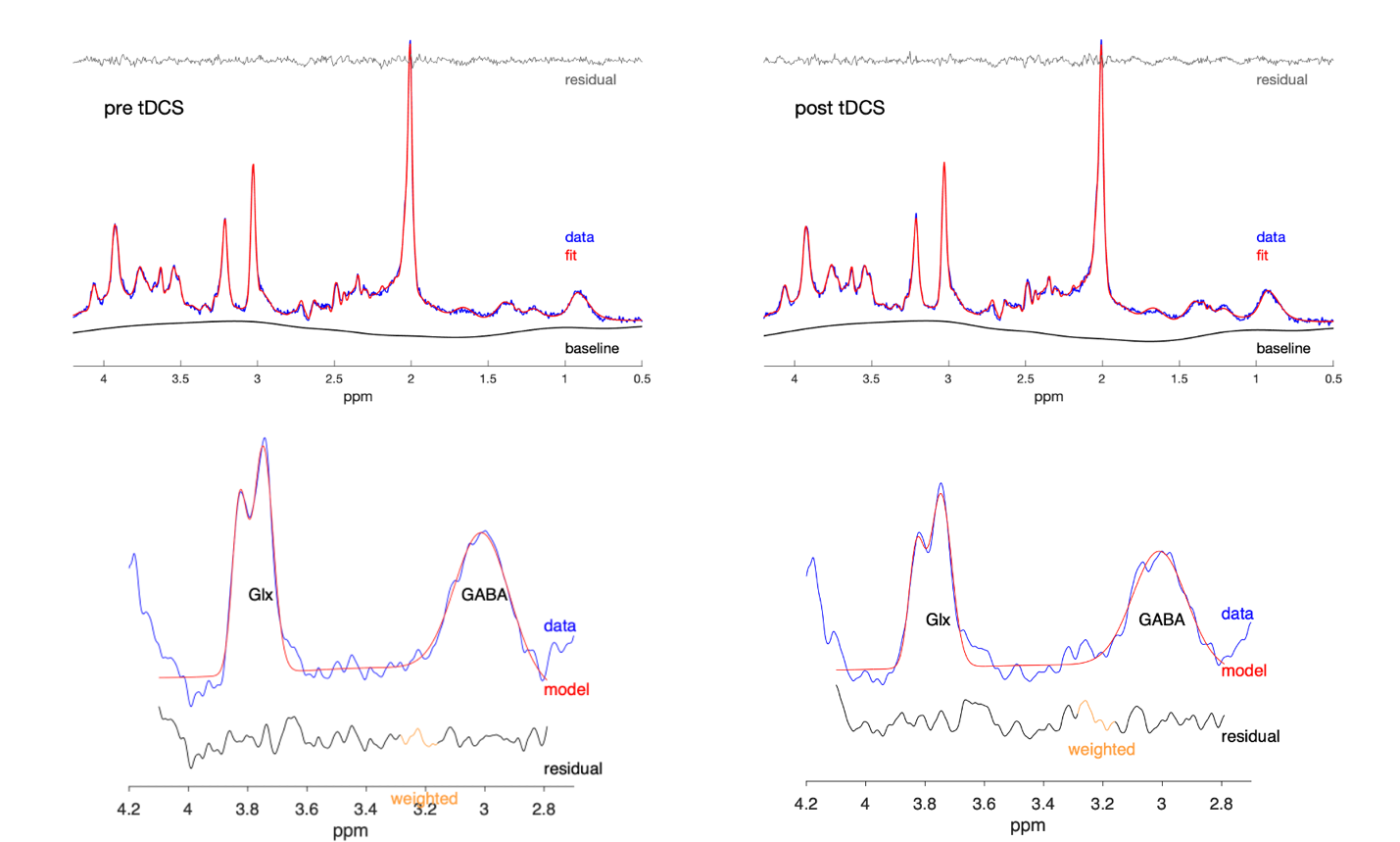


Supplementary figure 19: Example pre (left) and post (right) SemiLASER (top) and MEGA-PRESS (bottom) fits.
